# Evaluation of a chiral cubane-based Schiff base ligand in asymmetric catalysis reactions

**DOI:** 10.3762/bjoc.8.207

**Published:** 2012-10-22

**Authors:** Kyle F Biegasiewicz, Michelle L Ingalsbe, Jeffrey D St. Denis, James L Gleason, Junming Ho, Michelle L Coote, G Paul Savage, Ronny Priefer

**Affiliations:** 1 Department of Chemistry, Biochemistry, and Physics, Niagara University, NY 14109, USA; 2 Department of Chemistry, McGill University, Montreal, QC, H3A 2K6, Canada; 3 ARC Centre of Excellence for Free-Radical Chemistry and Biotechnology, Research School of Chemistry, Australian National University, Canberra ACT 0200, Australia; 4 CSIRO Materials Science and Engineering, Clayton South MDC 3169, Australia; 5 College of Pharmacy, Western New England University, Springfield, MA 01119, USA

**Keywords:** chiral ligand, cubane, M06L and B3LYP calculations, Michael addition

## Abstract

Recently, a novel chiral cubane-based Schiff base ligand was reported to yield modest enantioselectivity in the Henry reaction. To further explore the utility of this ligand in other asymmetric organic transformations, we evaluated its stereoselectivity in cyclopropanation and Michael addition reactions. Although there was no increase in stereocontrol, upon computational evaluation using both M06L and B3LYP calculations, it was revealed that a pseudo six-membered ring exists, through H-bonding of a cubyl hydrogen to the copper core. This decreases the steric bulk above the copper center and limits the asymmetric control with this ligand.

## Introduction

Since the initial synthesis of cubane in 1964 by Eaton and Cole [1–2], numerous studies have been undertaken on its derivatives: Nitrated cubanes are remarkably explosive [3–4]; cubylamines possess antiviral activity [5]; and cubylamides have been shown to be P2X7 receptor antagonists [6]; whereas other cubane derivatives were examined as narcotic antagonists against both μ and ĸ receptors [7], as well as being monoamine oxidase (MAO) inactivators [8–10]. Cubanes have also shown a propensity to undergo cage opening. In particular, *syn*-tricyclooctadienes are formed when rhodium(I) salts are introduced [11], while with silver(I) or palladium(II) catalysts, cuneane is obtained [12]. Spontaneous cage opening has been observed with cubanol yielding vinylcyclobutenylketene [13–14], whereas dicubyl disulfide is remarkably stable [15]. More recently, 4-iodo-1-vinylcubane was shown to undergo cage opening/rearrangement to form 4-vinyl-*trans*-β-iodostyrene [16–17], whereas 1-iodocubane-4-carboxaldehyde undergoes cage opening/fragmentation to afford benzaldehyde, which further reacts to give benzyl benzoate [18].

**Figure 1:**
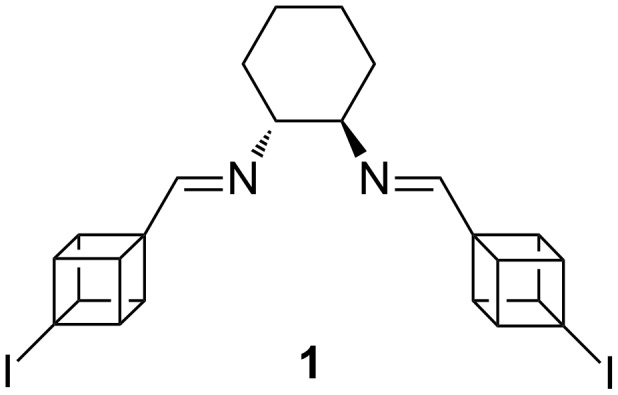
Structure of (1*R*,2*R*)-*N*,*N*’-bis[(4-iodocuban-1-yl)methylene]-*trans*-1,2-diamino cyclohexane (**1**).

Recently, we synthesized the first cubane-based Schiff base ligand (Figure 1) and screened it in the Henry reaction in the synthesis of β-nitroalcohols [19]. The cubyl moiety can be considered a cross between a *tert*-butyl and a phenyl group. In fact, the C–H bond of cubane has been shown to have ~31% s-character [20], compared to 25% for a simple alkane and 33% for an aromatic hydrogen. Due to the bulk of the cube it was initially envisioned that there would be high stereocontrol; however, the stereoselectivity was modest-at-best with only the highest ee value of 39% being achieved when copper(I) chloride was used at 65 °C. Therefore, we decided to examine this novel ligand further in the hope of increasing the stereoselectivity in other organic transformations.

## Results and Discussion

We began by initially evaluating this cubane-based ligand with cyclopropanation reactions. When no chiral ligand was added there was a 2.6:1 ratio of trans to cis products with no ee control. We then introduced our cubane-based chiral ligand **1** to our cyclopropanation protocol with four different copper sources (Table 1). The highest ee value of 14% was found with the use of Cu(I) triflate tetrakisacetonitrile as catalyst. For the reaction with Cu(I) chloride, values of only 1% ee were obtained for both cis and trans products. In all reactions, the trans product was favored over the cis. Overall, this reaction was unsuccessful in obtaining high stereoselectivity, and thus we decided to switch to Michael addition with organomagnesium and organozinc reagents.

**Table 1:** Cyclopropanation with cubane ligand **1**.

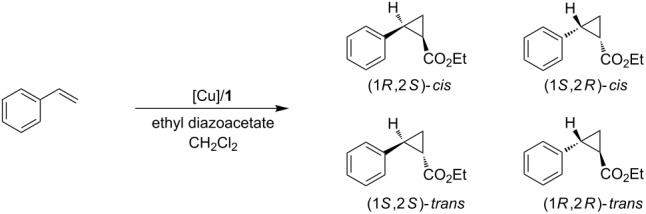					

Entry	Catalyst precursor	Conv (%)^a^	cis/trans^b^	ee^b^ cis (%)	ee^b^ trans (%)

1	Cu(OTf) toluene complex	53	45:55	9	5
2	Cu(OTf) tetrakisacetonitrile	13	29:71	14	9
3	CuCl	77	37:63	1	1
4	Cu(OTf)_2_	82	42:58	8	6

^a^The conversion was determined by analysis of the ^1^H NMR spectra. ^b^Determined by GC analysis using a Chirasil-Dextrin CB column.

Since the copper source that produced the highest ee value with the cyclopropanation above was Cu(I) triflate tetrakisacetonitrile, we decided to initially focus on this catalyst precursor. We were pleased to observe an ee value of 16% in the Michael addition of EtMgBr to 2-cyclohexen-1-one at 0 °C in Et_2_O (Table 2, entry 1). We next screened a variety of solvents in this reaction. With both CH_2_Cl_2_ and THF the ee values significantly dropped (Table 2, entry 2 and 3). As previously reported, CuCl gave the best results with the Henry reaction [19]; hence, we decided to evaluate this copper source. This, however, did not yield any encouraging results with only a maximum ee value of 4% when performed in Et_2_O (Table 2, entry 4). We next evaluated a Cu(OTf) toluene complex, as well as Cu(OTf)_2_; however, we only obtained a maximum value of 12% ee with Cu(OTf) _2_ in Et_2_O (Table 2, entry 10). The use of Et_2_Zn has also been used to provide excellent stereoselectivity in Michael addition reactions [21–22]. However, with our cubane-based chiral ligand **1** we did not have this success. Our best result with this anion source was with Cu(OTf)_2_ in Et_2_O reaching an ee value of only 12% (Table 2, entry 20).

**Table 2:** Asymmetric ethyl addition to 2-cyclohexen-1-one under different conditions.

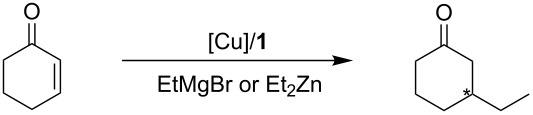					

Entry	Catalyst precursor	Alkylating reagent	Solvent	Conv (%)^a^	ee (%)^b^

1	Cu(OTf) tetrakisacetonitrile	EtMgBr	Et_2_O	>99	16 (*R*)
2	Cu(OTf) tetrakisacetonitrile	EtMgBr	CH_2_Cl_2_	89	<1 (*S*)
3	Cu(OTf) tetrakisacetonitrile	EtMgBr	THF	>99	3 (*R*)
4	CuCl	EtMgBr	Et_2_O	>99	4 (*S*)
5	CuCl	EtMgBr	CH_2_Cl_2_	92	1 (*S*)
6	CuCl	EtMgBr	THF	>99	2 (*R*)
7	Cu(OTf) toluene complex	EtMgBr	Et_2_O	>99	1 (*R*)
8	Cu(OTf) toluene complex	EtMgBr	CH_2_Cl_2_	>99	15 (*S*)
9	Cu(OTf) toluene complex	EtMgBr	THF	94	11 (*R*)
10	Cu(OTf)_2_	EtMgBr	Et_2_O	>99	12 (*S*)
11	Cu(OTf)_2_	EtMgBr	CH_2_Cl_2_	>99	5 (*S*)
12	Cu(OTf)_2_	EtMgBr	THF	>99	1 (*S*)
13	Cu(OTf) tetrakisacetonitrile	Et_2_Zn	Et_2_O	86	3 (*S*)
14	Cu(OTf) tetrakisacetonitrile	Et_2_Zn	CH_2_Cl_2_	87	9 (*R*)
15	CuCl	Et_2_Zn	Et_2_O	>99	9 (*S*)
16	CuCl	Et_2_Zn	CH_2_Cl_2_	>99	11 (*S*)
17	CuCl	Et_2_Zn	THF	>99	4 (*S*)
18	Cu(OTf) toluene complex	Et_2_Zn	Et_2_O	>99	4 (*S*)
19	Cu(OTf) toluene complex	Et_2_Zn	CH_2_Cl_2_	>99	1 (*S*)
20	Cu(OTf)_2_	Et_2_Zn	Et_2_O	>99	12 (*S*)
21	Cu(OTf)_2_	Et_2_Zn	CH_2_Cl_2_	97	6 (*S*)
22	Cu(OTf)_2_	Et_2_Zn	THF	>99	1 (*S*)

^a^The conversion was determined by analysis of the ^1^H NMR spectra. ^b^Determined by HPLC (OD Column, 0.5 mL/min, 99.7:0.3 hexanes/iPrOH) after derivatization with (*R*,*R*)-1,2-diphenylethane-1,2-diol.

At the onset we had anticipated much stronger results as the large bulky nature of the cube should likely block one side of the copper complex, thus hopefully increasing stereoselectivity. Appropriate crystals for X-ray analysis were not obtained, thus we decided to look into computational simulations. On the basis of earlier assessment studies [23–24], we chose the M06L [25] density functional theory method (and B3LYP [26] for comparison) to locate the global minimum-energy structures for the ligands and their Cu(I) complexes. The computations employed the 6-31+G(d) basis set, and for iodine the LAN2DZdp basis set [27] with an effective core potential was used instead of 6-31+G(d). All calculations were performed with Gaussian09 [28].

**Figure 2:**
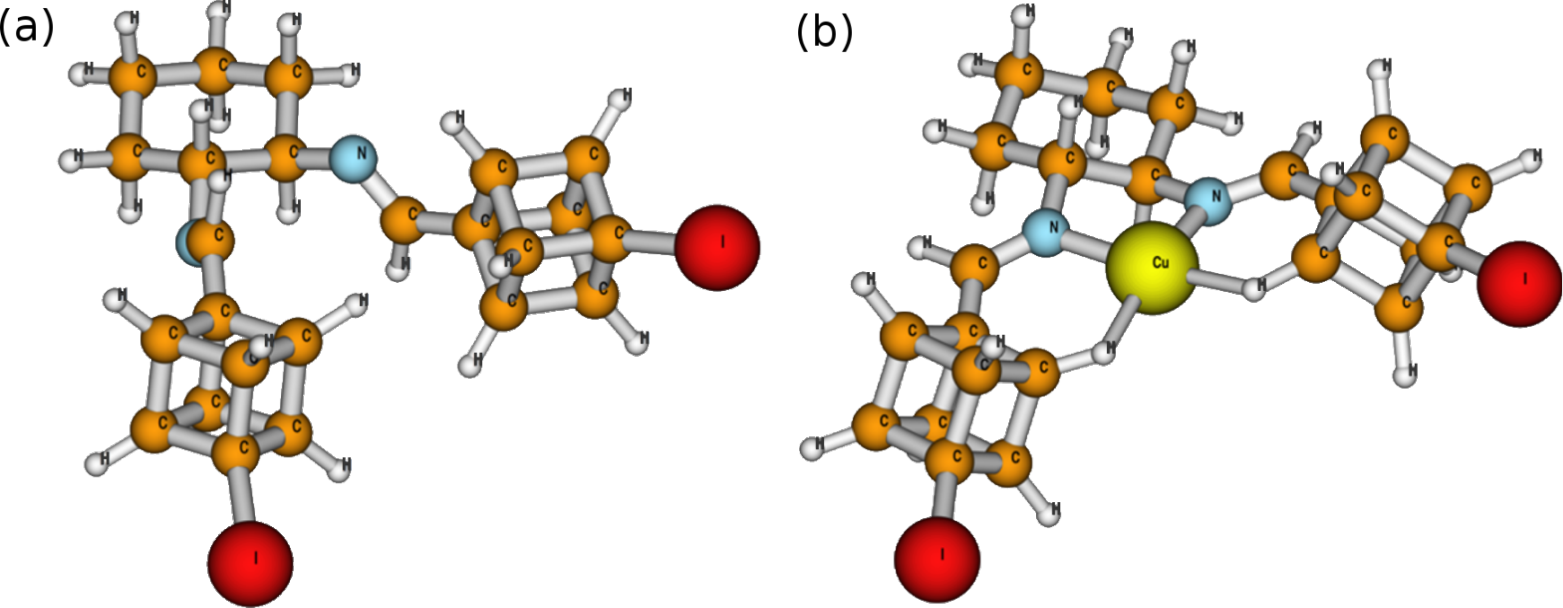
M06L DFT global minima for (a) (1*R*,2*R*)-*N*,*N*’-bis[(4-iodocuban-1-yl)methylene]-*trans*-1,2-diaminocyclohexane (**1**) and (b) Cu^+1^ complex with ligand **1**.

Using both B3LYP and M06L density functional theory methods, we observed N–C–C–N dihedral angles of 65.0 and 62.7°, respectively for the ligand **1** on its own (Figure 2a). When this was complexed with a Cu^+1^, however, the cyclohexyl moiety retained its chair conformation, but the N–C–C–N dihedral angles dropped to 49.7° and 48.5°, in the B3LYP and M06L calculations, respectively (Figure 2b). In addition, the copper is observed to exhibit either an agostic interaction [29] or a H-bond [30] with the “meta” cubyl hydrogens (with respect to the iodine). The H^…^Cu bond lengths of 2.06 Å with M06L and 2.29 Å with B3LYP calculations are well within the required H-bonding length of *<*3.2 Å [31]. In addition, the C^…^Cu lengths of 2.85 and 3.03 Å with M06L and B3LYP, respectively, were well within the required 4.0 Å limit [31]. This produces pseudo six-membered rings having a C–H^…^Cu bond angle of 125.1° or 122.9° with M06L and B3LYP calculations, respectively. As Cu(I) is a late transition metal in a low oxidation state [32], the H^…^Cu bond length is shorter than that of C^…^Cu [27], and the C–H^…^Cu bond angle is >100° [33], it can be stated that the H^…^Cu interaction is a true intermolecular multicenter hetero-acceptor hydrogen bond as opposed to an intermolecular pseudo-agostic bond. Cubyl hydrogens participating in H-bonding have been reported previously with nitrocubanes [34], as well as with dicubyl *vic*-disulfone [35]. From the computational results it would appear that the cubes play very little role in blocking either side for selective coordination with the copper. In fact, it would appear that the very modest selectivity is due to the axial hydrogens on the cyclohexyl moiety since the copper is best described as being strained square planar with a N–Cu–H bond angle of 93.7° for M06L and 91.0° for B3LYP.

## Conclusion

We obtained slight stereoselectivity for both cyclopropanation as well as Michael addition. A maximum ee value of 16% was observed when Michael addition was performed with EtMgBr at 0 °C in Et_2_O. The poor stereoselectivity is explained computationally by the lack of steric hindrance on either face of the copper complex. In addition, H-bonding with cubyl hydrogens and the copper core yielded a pseudo six-membered ring, which decreased the N–C–C–N dihedral angle.

## Supporting Information

File 1 Gaussian archives of (1*R*,2*R*)-*N*,*N*’-bis[(4-iodocuban-1-yl)methylene]-*trans*-1,2-diaminocyclohexane (**1**) and Cu^+1^ complex with ligand **1**.

## References

[1] Eaton P E, Cole T W (1964). J Am Chem Soc.

[2] Eaton P E, Cole T W (1964). J Am Chem Soc.

[3] Eaton P E, Zhang M-X, Gilardi R, Gelber N, Iyer S, Surapaneni R (2002). Propellants, Explos, Pyrotech.

[4] Zhang M-X, Eaton P E, Gilardi R (2000). Angew Chem, Int Ed.

[5] Camps P, Duque M D, Vázquez S, Naesens L, De Clercq E, Sureda F X, López-Querol M, Camins A, Pallàs M, Prathalingam S R (2008). Bioorg Med Chem.

[6] Gunosewoyo H, Guo J L, Bennett M R, Coster M J, Kassiou M (2008). Bioorg Med Chem Lett.

[7] Cheng C-Y, Hsin L-W, Lin Y-P, Tao P-L, Jong T-T (1996). Bioorg Med Chem.

[8] Zhou J J P, Li J, Upadhyaya S, Eaton P E, Silverman R B (1997). J Med Chem.

[9] Silverman R B, Zhou J J P, Eaton P E (1993). J Am Chem Soc.

[10] Silverman R B (1995). Acc Chem Res.

[11] Cassar L, Eaton P E, Halpern J (1970). J Am Chem Soc.

[12] Eaton P E, Cassar L, Halpern J (1970). J Am Chem Soc.

[13] Cole T W (1966).

[14] Hormann R E (1987).

[15] Priefer R, Lee Y J, Barrios F, Wosnick J H, Lebuis A-M, Farrell P G, Harpp D N, Sun A, Wu S, Snyder J P (2002). J Am Chem Soc.

[16] Carroll V M, Harpp D N, Priefer R (2008). Tetrahedron Lett.

[17] Griffiths J R, Tsanaktsidis J, Savage G P, Priefer R (2010). Thermochim Acta.

[18] Heaphy P J, Griffiths J R, Dietz C J, Savage G P, Priefer R (2011). Tetrahedron Lett.

[19] Ingalsbe M L, St Denis J D, Gleason J L, Savage G P, Priefer R (2010). Synthesis.

[20] Della E W, Hine P T, Patney H K (1977). J Org Chem.

[21] Shibata N, Yoshimura M, Yamada H, Arakawa R, Sakaguchi S (2012). J Org Chem.

[22] Matsumoto Y, Yamada K-i, Tomioka K (2008). J Org Chem.

[23] Zhao Y, Truhlar D G (2008). Theor Chem Acc.

[24] Zhao Y, Truhlar D G (2008). Acc Chem Res.

[25] Zhao Y, Truhlar D G (2006). J Chem Phys.

[26] Becke A D (1993). J Chem Phys.

[27] Wadt W R, Hay P J (1985). J Chem Phys.

[28] (2009). Gaussian 09.

[29] Brookhart M, Green M L H (1983). J Organomet Chem.

[30] Thakur T S, Desiraju G R (2006). Chem Commun.

[31] Jeffrey G A (1997). An Introduction to Hydrogen Bonding.

[32] Braga D, Grepioni F, Desiraju G R (1998). Chem Rev.

[33] Braga D, Grepioni F, Tedesco E, Biradha K, Desiraju G R (1997). Organometallics.

[34] Zhou G, Zhang J-L, Wong N-B, Tian A (2003). J Mol Struct.

[35] Priefer R, Martineau E, Harpp D N (2007). J Sulfur Chem.

