# Supplementary material for: Evaluation of a chiral cubane-based Schiff base ligand in asymmetric catalysis reactions
Source: Beilstein J Org Chem. 2012 Oct 22;8:1814–8. doi: 10.3762/bjoc.8.207 (PMC3511016; doi:10.3762/bjoc.8.207)
Supplement: File 1 — Gaussian archives of (1R,2R)-N,N’-bis[(4-iodocuban-1-yl)methylene]-trans-1,2-diaminocyclohexane (1) and Cu+1 complex with ligand 1. [file Beilstein_J_Org_Chem-08-1814-s001.pdf]

Supporting Information

for

Evaluation of a chiral cubane-based Schiff base ligand in asymmetric catalysis reactions

Kyle F. Biegasiewicz[1], Michelle L. Ingalsbe[1], Jeffrey D. St. Denis[2], James L. Gleason[2], Junming Ho[3], Michelle L. Coote[3], G. Paul Savage[4], Ronny Priefer*[1,5]

Address: [1]Department of Chemistry, Biochemistry, and Physics, Niagara University, NY 14109, USA, [2]Department of Chemistry, McGill University, Montreal, QC, H3A 2K6, [3]ARC Centre of Excellence for Free-Radical Chemistry and Biotechnology, Research School of Chemistry, Australian National University, Canberra ACT 0200, Australia, [4]CSIRO Materials Science and Engineering, Clayton South MDC 3169, Australia and [5]College of Pharmacy, Western New England University, Springfield, MA 01119, USA

E-mail: Ronny Priefer - ronny.priefer@wne.edu

*Corresponding author

Gaussian archives of (1*R*,2*R*)-*N*,*N*'-bis[(4-iodocuban-1-yl)methylene]-*trans*-1,2-diaminocyclohexane (1) and Cu^{+1} complex with ligand 1.

Table of contents

Synthesis of (1*R*,2*R*)-*N*,*N*'-bis[(4-iodocuban-1-yl)methylene]-*trans*-1,2-diaminocyclohexane (**1**)........ S2
General Procedure of cyclopropanation........ S3
General Procedure of Michael Addition........ S3
The B3LYP gas phase optimized geometries of cubane ligand **1** S4
The B3LYP gas phase optimized geometries of Cu^{+1} complex with cubane ligand **1**........ S5
The M06L gas phase optimized geometries of cubane ligand **1**........ S7
The M06L gas phase optimized geometries of Cu^{+1} complex with cubane ligand **1**........ S8

NOTE: All species had zero imaginary frequencies, as determined from frequency calculations at the respective levels of theory

Synthesis of (1*R*,2*R*)-*N*,*N*'-bis[(4-iodocuban-1-yl)methylene]-*trans*-1,2-diaminocyclohexane (1)

trans-(1*R*,2*R*)-(−)-1,2-Cyclohexanediamine (50.3 mg, 0.438 mmol) and 1-iodocubane-4-carboxaldehyde (226 mg, 0.876 mmol) and oven-dried 4 Å molecular sieves (0.05 g) were combined together in ethanol (1.1 mL) at RT and stirred for 30 min. The mixture was evaporated to dryness, affording an off-white solid. The solid was collected and washed with cold ethanol under vacuum filtration, yielding a white solid (195 mg, 75.0%). Mp 95–97 °C; ^{1}H NMR (400 MHz, $CDCl_3$) δ 1.36–1.40 (m, 2H), 1.61–1.84 (m, 6H), 3.06–3.09 (m, 2H), 4.21–4.32 (m, 6H), 7.66 (s, 2H); ^{13}C NMR (100 MHz, $CDCl_3$) δ 162.0, 73.7, 59.0, 54.7, 49.9, 37.5, 32.6, 24.4; HRMS–ESI (*m*/*z*): $[M + H]^+$ calcd for $C_{24}H_{25}N_2I_2$, 595.0102; found, 595.01074.

General Procedure of cyclopropanation

The copper catalyst (0.00841 mmol) and cube ligand (**1**, 10 mg, 0.0168 mmol) were added to a flame-dried round-bottom flask at ambient temperature. Styrene (53.2 µL, 0.463 mmol) was added by syringe, and anhydrous dichloromethane (600 µL) was added as the solvent. The mixture was stirred for 30 min to homogenize. 1 M Ethyl diazoacetate (43.6 µL in 421 µL CH_2Cl_2, 0.421 mmol) was added over a period of 4 h by using a syringe pump followed by work-up with EtOAc, washing with brine, drying, filtering, and evaporation.

General Procedure of Michael addition

A round-bottom flask was charged with a copper source (0.05 equiv), cube ligand (0.1 equiv), and solvent and stirred for 30 min. Cyclohexen-1-one (1 equiv) was added and the reaction mixture was cooled to 0 °C. Finally, the alkylating reagent (1.3 equiv) was added and the reaction mixture held at <0 °C for 15 h. The reaction mixture was quenched with NH_4Cl, and the product was extracted with Et_2O, dried, and concentrated under reduced pressure to yield 3-cyclohexanone.

B3LYP gas phase optimized geometries of cubane ligand **1**

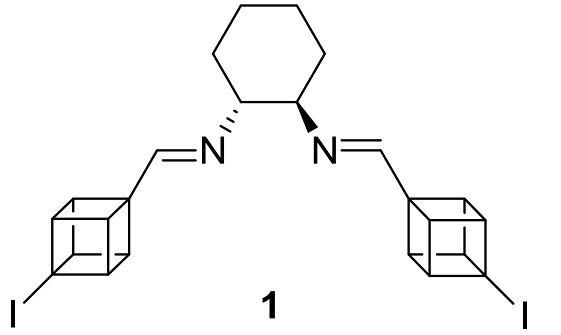

1

N	-0.1175485228	0.2220152356	-0.7577500142
N	-2.2083115496	-1.5078278677	0.3395789442
C	0.4121396518	-0.4150870167	-1.7257522813
H	-0.1838470553	-0.7349422701	-2.60007149
C	-1.6898853912	-2.1259612812	1.3258054053
H	-1.2813035918	-1.5816674178	2.1968660922
C	1.8433079321	-0.770064053	-1.7596421946
C	2.4110578968	-2.2446100725	-1.889085591
H	1.798297688	-3.1467612472	-1.9376548944
C	3.4158888211	-1.7708315855	-2.9945780451
H	3.6354059339	-2.2988264549	-3.9227097927
C	2.8639472507	-0.3082567438	-2.8825830577
H	2.6137715684	0.3396431224	-3.7249033188
C	2.9070587386	-0.4521366265	-0.6569554716
H	2.6904042487	0.0759477724	0.2711308376
C	3.4586395461	-1.9181340898	-0.7673258387
H	3.7106615892	-2.5682124002	0.0708591013
C	4.4596968145	-1.4397357215	-1.8741100358
C	3.9127137079	0.0247183007	-1.764106951
H	4.5249000625	0.9256276405	-1.7208685251
I	6.5381719707	-1.9653638995	-1.9503464913
C	-1.5965983021	-3.5966922718	1.3862019405
C	-2.0835187852	-4.6054393657	0.2935449132
C	-2.2491679703	-4.4993046898	2.5155426519
H	-2.8539694349	-4.1232725532	3.3429969114
C	-0.249081378	-4.4158953527	1.5489472337
H	0.7471729397	-3.9730218235	1.6041845797
C	-2.7479925515	-5.4908974396	1.4066287152
H	-3.7429988184	-5.9333365606	1.3567339694
H	-2.551136912	-4.3140297776	-0.6464392554
C	-0.7412535553	-5.4075371063	0.4368447199
H	-0.134697509	-5.7857033355	-0.3864116068
C	-1.406419864	-6.2882029554	1.549469255

C	-0.9103576407	-5.3011194719	2.6604132793
H	-0.4433392322	-5.5953286742	3.6004822328
I	-1.2613912179	-8.4254725053	1.6643671078
C	-2.2727808378	-0.0525601217	0.3905143339
C	-1.7003948501	2.0763106073	-0.8719999923
C	-3.9087280954,	1.9081334427	0.3543970534
C	-3.1745519191	2.5036033247	-0.855288244
C	-3.7517225187	0.3819240801	0.3994708072
C	-1.5370573271	0.543979391	-0.8344882232
H	-1.1742496556	2.5032730131	-0.0067798366
H	-3.5038793348	2.3478984201	1.2782498602
H	-3.6662789451	2.1678849134	-1.7806516555
H	-4.2533432022	-0.0744092063	-0.465322926
H	-1.9956651737	0.1086663441	-1.741059772
H	-1.7755907759	0.336546351	1.2977567349
H	-1.1954839474	2.4626359245	-1.7667151174
H	-4.9741715789	2.1706391721	0.3241969164
H	-3.243060883	3.5991341287	-0.84480482
H	-4.2347112704	-0.0306554968	1.2945671059

B3LYP gas phase optimized geometries of Cu^{+1} complex with cubane ligand **1**

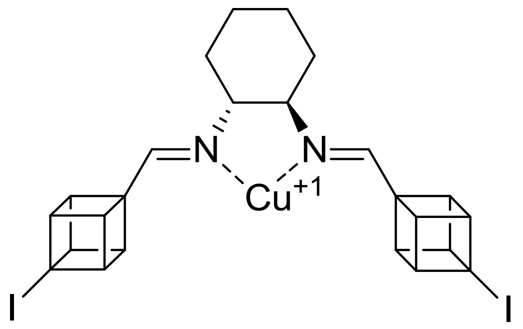

Cu	-0.0000014814	1.013246	0.0000005612
N	1.35032	2.443253	-0.357842
C	0.601322	3.718472	-0.501931
C	-0.601354	3.718467	0.501917
N	-1.350369	2.443259	0.357818
C	-1.439153	4.998169	0.36432
C	-0.576139	6.261826	0.504297
C	0.57613	6.261828	-0.504274
C	1.439134	4.998161	-0.364313
C	2.63047	2.361843	-0.385606
C	3.337254	1.085031	-0.251093

C	-2.630515	2.361864	0.385594
C	-3.3373	1.085048	0.251087
C	-4.443323	0.776891	-0.848798
C	-3.790477	-0.628877	-1.080783
C	-4.734652	-1.229472	0.020811
C	-3.663134	-0.9266	1.128393
C	-2.71687	-0.3354	0.025107
C	-5.387106	0.180546	0.248597
C	-4.316651	0.479743	1.350748
C	2.716847	-0.335435	-0.025155
C	3.663166	-0.926594	-1.128419
C	4.316659	0.479767	-1.350727
C	5.387082	0.180575	-0.248543
C	4.443247	0.776879	0.848828
C	4.734651	-1.229462	-0.020804
C	3.790423	-0.628906	1.080765
I	-5.799154	-3.060173	-0.164711
I	5.799189	-3.060141	0.164714
H	3.254454	3.252018	-0.515893
H	-1.644684	-0.576979	-0.067472
H	-3.360791	-1.610888	1.920337
H	-4.510549	0.939204	2.320878
H	-4.740019	1.472004	-1.635507
H	-3.590153	-1.075381	-2.054142
H	-6.455597	0.37185	0.33631
H	4.510579	0.939249	-2.320842
H	1.644666	-0.577052	0.067378
H	6.455573	0.371903	-0.336214
H	4.739896	1.471987	1.635559
H	3.590074	-1.075427	2.054112
H	3.360863	-1.610873	-1.920386
H	-1.941319	5.001882	-0.614388
H	0.17421	6.321153	-1.52587
H	-0.174218	6.321135	1.525894
H	1.941296	5.001857	0.614398
H	-0.169232	3.686204	1.513674
H	0.169204	3.686226	-1.51369
H	-2.224371	5.011745	1.129426
H	1.206393	7.147576	-0.366478
H	-1.206394	7.147582	0.366511
H	2.224355	5.011741	-1.129416
H	-3.254494	3.252043	0.515885

M06L gas phase optimized geometries of cubane ligand **1**

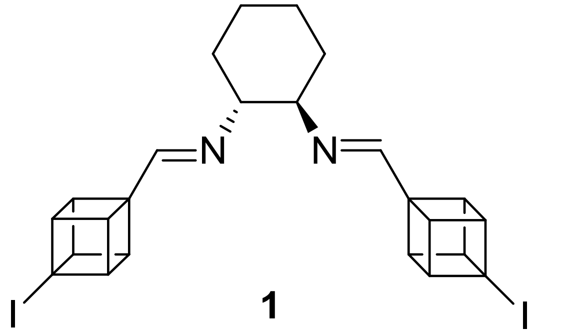

1

C	0.3832678533	-0.5860861897	4.3316072288
C	0.1061419431	-1.454094409	5.558212936
C	0.4099500079	-0.7128956006	6.8535281384
C	-0.3748975083	0.5913006075	6.9334289412
C	-0.1068917982	1.4625895063	5.7136458525
C	-0.4209027189	0.7267088706	4.4144541559
N	-0.0744601618	1.5507146282	3.2743135913
C	-0.9328118455	1.6349058365	2.3359758702
C	-0.6590847843	2.3378716983	1.081106503
C	0.6339951228	3.1091843435	0.7284928325
N	-0.0013116725	-1.3008860553	3.1309653194
C	0.8964859988	-1.4992892699	2.2482924525
C	0.6309534021	-2.2310897791	1.0054874721
C	-0.7591950593	-2.6679236863	0.4843611328
C	1.1998964342	-3.6610624459	0.6902065775
C	1.5964883368	-3.1975076806	-0.7330716764
C	1.0423929834	-1.7806330487	-0.4353578075
C	-0.3630873907	-2.2018091401	-0.9432589139
C	-0.2216990489	4.242337266	0.1006661512
C	-1.5253569566	3.4675160087	0.4303192682
C	-1.46470143	2.7396076351	-0.9369987244
C	-0.6109914797	1.6108085666	-0.3072780845
C	-0.1595418015	3.508533733	-1.2628841651
C	0.696221952	2.3764374173	-0.6389111496
C	-0.2040918925	-4.0876543741	0.1903168463
C	0.1918923211	-3.6189028687	-1.233236133
I	0.2594674463	4.4527548826	-3.1425127283
I	-0.1816479483	-4.7256716071	-3.0306646178
H	-1.9219404439	1.1352176644	2.4098524275
H	1.9382749049	-1.1420572823	2.3938185729
H	-0.7748765184	0.5358922595	-0.4441421557
H	-2.3033878343	2.5977819991	-1.6235333371
H	-2.4244065802	3.9047760665	0.8763024643
H	1.4717324879	3.2478822197	1.4165737462

H	1.5920976733	1.9403851562	-1.0897204462
H	-0.0682417305	5.3139237332	0.2525524709
H	1.8328818261	-4.2401542125	1.3699281407
H	1.5477488142	-0.8396739276	-0.6781771896
H	-0.7024110781	-5.029700644	0.4328789957
H	-1.7013505771	-2.4448363458	0.9926526976
H	-0.9924251922	-1.6213442845	-1.6242516134
H	2.540764715	-3.4202820746	-1.2364062882
H	0.950531748	1.7682740365	5.6903445868
H	1.4880272536	-0.4909228813	6.9044811878
H	-1.4514124933	0.363539884	6.9926277504
H	-0.9515506164	-1.7586165466	5.533837859
H	-1.4949854127	0.4457186632	4.3964564481
H	1.4594640723	-0.3129798238	4.31039557
H	-0.6942247505	2.3896379859	5.7530981004
H	0.1861723914	-1.3547388395	7.7158141481
H	-0.1248359118	1.1387497308	7.851696752
H	0.6952689476	-2.3782163816	5.4847538426

M06L gas phase optimized geometries of Cu^{+1} complex with cubane ligand **1**

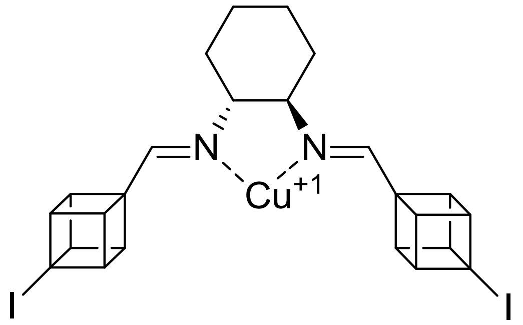

Cu	0.	0.	1.0788714843
N	0.5107178368	-1.273358628	2.5375758803
C	0.5643071503	-0.5345324784	3.809865899
C	-0.5643071503	0.5345324784	3.809865899
N	-0.5107178368	1.273358628	2.5375758803
C	-0.5113076738	1.3823418557	5.0727006339
C	-0.5603535097	0.5144843836	6.3251139659
C	0.5603535097	-0.5144843836	6.3251139659
C	0.5113076738	-1.3823418557	5.0727006339
C	0.6006774412	-2.5501692257	2.4510802128
C	0.510266261	-3.214401033	1.1555391889
C	-0.6006774412	2.5501692257	2.4510802128
C	-0.510266261	3.214401033	1.1555391889
C	0.5004106306	4.3605399487	0.7959068074
C	0.7979934722	3.6483740443	-0.5482814626

C	-0.3472945704	4.4615879431	-1.2112756237
C	-1.3724933133	3.3485511266	-0.8580118371
C	-0.2247152609	2.534406902	-0.2053580453
C	-0.6386543573	5.1755456027	0.1369126959
C	-1.655308058	4.0625069347	0.4869082914
C	0.2247152609	-2.534406902	-0.2053580453
C	1.3724933133	-3.3485511266	-0.8580118371
C	1.655308058	-4.0625069347	0.4869082914
C	0.6386543573	-5.1755456027	0.1369126959
C	-0.5004106306	-4.3605399487	0.7959068074
C	0.3472945704	-4.4615879431	-1.2112756237
C	-0.7979934722	-3.6483740443	-0.5482814626
I	-0.2080132213	5.4167822254	-3.1035947437
I	0.2080132213	-5.4167822254	-3.1035947437
H	0.7440377113	-3.1819712004	3.3395442177
H	-0.0531335407	1.4456905222	-0.3841820186
H	-2.1333054211	2.9472524669	-1.5311976671
H	-2.6444442999	4.2126652858	0.9284279245
H	1.2545480007	4.7548448202	1.4831385904
H	1.7928229157	3.4902401934	-0.9706885129
H	-0.8054334841	6.2472993023	0.2595780801
H	2.6444442999	-4.2126652858	0.9284279245
H	0.0531335407	-1.4456905222	-0.3841820186
H	0.8054334841	-6.2472993023	0.2595780801
H	-1.2545480007	-4.7548448202	1.4831385904
H	-1.7928229157	-3.4902401934	-0.9706885129
H	2.1333054211	-2.9472524669	-1.5311976671
H	0.4157141988	1.9787779581	5.0684689158
H	1.5328791501	-0.0014243929	6.3760900223
H	-1.5328791501	0.0014243929	6.3760900223
H	-0.4157141988	-1.9787779581	5.0684689158
H	-1.5203719635	-0.0173349467	3.7867471323
H	1.5203719635	0.0173349467	3.7867471323
H	-1.3461531657	2.0952653884	5.0786791317
H	0.5023500951	-1.149413239	7.2165675786
H	-0.5023500951	1.149413239	7.2165675786
H	1.3461531657	-2.0952653884	5.0786791317
H	-0.7440377113	3.1819712004	3.3395442177
